# Response of Nitrogen Cycling in Alfalfa (*Medicago sativa* L.) Grassland Systems to Cropping Patterns and Nitrogen Application Rates: A Quantitative Analysis Based on Nitrogen Balance

**DOI:** 10.3390/plants14233647

**Published:** 2025-11-29

**Authors:** Yaya Duan, Jianxin Yin, Yuanbo Jiang, Haiyan Li, Wenjing Chang, Yanbiao Wang, Minhua Yin, Yanxia Kang, Yanlin Ma, Yayu Wang, Guangping Qi

**Affiliations:** 1 College of Water Conservancy and Hydropower Engineering, Gansu Agricultural University, Lanzhou 730070, China; 1073323020376@st.gsau.edu.cn (Y.D.); 1073323010121@st.gsau.edu.cn (Y.J.); 107332201100@st.gsau.edu.cn (H.L.); 1073324120789@st.gsau.edu.cn (W.C.); 1073323020378@st.gsau.edu.cn (Y.W.); mayl@gsau.edu.cn (Y.M.); wangyy@gsau.edu.cn (Y.W.); qigp@gsau.edu.cn (G.Q.); 2 Zhejiang Institute of Quality Sciences, Hangzhou 310018, China; 18834171849@163.com

**Keywords:** ridge cultivation with plastic mulch, nitrogen transport, nitrogen uptake, greenhouse gases, nitrogen balance, alfalfa

## Abstract

An imbalance between the supply and demand of nutrients within the crop–soil system has resulted from the prevalent practice of excessive fertilization in agricultural agriculture. In order to increase crop growth, improve resource usage efficiency, and reduce agricultural nonpoint source pollution, appropriate cropping management techniques are essential. This study examined the effects of four nitrogen application rates (0 kg·ha^−1^ (C0), 80 kg·ha^−1^ (C1), 160 kg·ha^−1^ (C2), and 240 kg·ha^−1^ (C3)) and three alfalfa cropping systems (traditional flat planting, FP; ridge-covered biodegradable mulch, JM; and ridge-covered conventional mulch, PM) on soil inorganic nitrogen transport, nitrogen allocation within alfalfa plants, and soil N_2_O emissions. Throughout the alfalfa growth phase, the dynamics of nitrogen balance within the soil–plant–atmosphere system were quantitatively examined. The findings showed: (1) The concentrations of soil NO_3_^−^–N and NH_4_^+^–N rose with the rate of nitrogen application but decreased with soil depth. The PMC3 treatment had the largest inorganic nitrogen reserves at the end of the alfalfa growth period. (2) The pattern of PM > JM > FP for nitrogen uptake and nitrogen accumulation in biomass in alfalfa leaves and stems peaked at the C2 nitrogen treatment rate. (3) As nitrogen application rates increased, grass-land N_2_O emission flow and total emissions also followed PM > JM > FP. (4) The PMC2 treatment showed apparent nitrogen balances of 9.73 kg·ha^−1^ and 1.84 kg·ha^−1^ during the two-year growing season, with apparent nitrogen loss rates of 6.08% and 1.15%, respectively, both significantly lower than other treatments, according to nitrogen balance analysis. In summary, the nitrogen application pattern combining ridge-covering conventional plastic mulch with moderate nitrogen application levels can achieve nitrogen balance in alfalfa grassland systems within the Yellow River irrigation district of Gansu Province, China, and similar ecological zones.

## 1. Introduction

As an essential macronutrient, nitrogen cycles through the soil–crop–atmosphere system via fixation, nitrification, and denitrification, which is critical for sustaining agroecosystem balance [1,2]. Yet, agricultural output does not increase linearly with nitrogen input [1,2]. When nitrogen fertilizer is applied in excess, large amounts of unutilized reactive nitrogen (Nr) are released into the environment, resulting in severe environmental impacts including nitrate leaching, groundwater pollution, water eutrophication, and substantial greenhouse gas emissions [3,4]. Agriculture is statistically the dominant source of anthropogenic N_2_O emissions globally [5]. Given that the average farm-level nitrogen use efficiency (NUE) worldwide is a mere 30–50%, nitrogen loss incurs over USD 100 billion in direct economic losses and environmental remediation costs annually [5,6,7]. Consequently, elucidating the key processes of nitrogen migration, transformation, and loss within this system is a pivotal step toward achieving nitrogen reduction, enhanced efficiency, and sustainable agriculture [8].

The optimization of cropping systems and nitrogen application strategies is an effective approach to improve nitrogen cycling within the soil–crop–atmosphere system [9]. Compared with traditional flat-bed cultivation, ridge-and-film mulching regulates the distribution of water and heat resources in farmland and modulates nitrogen transformation processes by altering microtopography [10]. This practice has been widely adopted in arid and semi-arid agricultural production [11]. Research has indicated that ridge-and-film cultivation effectively retains water and soil moisture, increases soil temperature and microbial activity, enhances soil nutrient availability, and creates favorable conditions for crop growth [12,13,14]. Currently, agricultural plastic mulch coverage in China spans 1840 ha, encompassing nearly all grain and cash crops [15]. However, the regulatory effects of ridge-mulching on soil nitrogen cycling remain complex and controversial. Research has indicated that the high-temperature, high-humidity environment created by plastic mulch significantly promotes the enrichment of ammonia-oxidizing bacteria (AOB), accelerating nitrification and leading to soil nitrate accumulation. This, in turn, increases the risk of nitrogen leaching and ammonium volatilization [16]. Simultaneously, the high-temperature, high-humidity conditions stimulate denitrification, particularly after rainfall or irrigation when soil aeration decreases, resulting in significantly increased N_2_O emissions [17,18]. Full-season plastic mulching can cause excessive soil temperatures during the late crop growth stage, accelerating root senescence [19]. Conversely, studies have indicated that the elevated soil thermal environment under ridge-planting mulching promotes early crop emergence and earlier canopy closure, reducing sub-mulch temperatures and soil moisture evaporation. This, in turn, decreases inorganic nitrogen volatilization and denitrification rates [16]. Additionally, crops enter the rapid nitrogen uptake phase earlier, enabling the early transfer of soil inorganic nitrogen into plant reserves. This reduces the window period during which nitrogen persists in the soil as readily leachable forms [20].

Nitrogen fertilizer application serves as a primary nitrogen source in agricultural systems and plays a critical role in enhancing farmland productivity [21]. Previous studies have demonstrated that appropriate external nitrogen supplementation supplies sufficient nutrients for soil microorganisms, promotes organic matter accumulation and nutrient equilibrium, improves soil fertility, and enhances crop uptake and utilization of phosphorus and potassium [22,23]. Additionally, nitrogen facilitates the synthesis of proteins, amino acids, and chlorophyll in crops. Through the regulation of endogenous hormones, it promotes crop growth and development [24,25] while improving stress tolerance and resource use efficiency [26,27]. However, crops exhibit physiological upper limits for nitrogen uptake across different growth stages. When external nitrogen inputs exceed the carrying capacity threshold of the crop–soil system, the carbon-nitrogen stoichiometric ratio in the rhizosphere is reduced, microbial carbon utilization is inhibited, the microbial pathway for organic matter formation is weakened, and microorganisms transition from “carbon limitation” to “nitrogen saturation” [28,29]. This disruption compromises efficient nitrogen cycling within the soil–microbe–plant continuum [28,29]. Moreover, elevated nitrogen concentrations cause substrate saturation in key nitrogen assimilation enzymes in crops, thereby reducing protein synthesis efficiency [30]. This not only diminishes nitrogen use efficiency (NUE) but also impedes the allocation of photosynthetic products to roots via osmotic stress, consequently impairing root nutrient uptake capacity [30,31].

A substantial body of research has hitherto been dedicated to the examination of the impact of individual factors on the yield of grain and cash crops. The focus of these studies has predominantly encompassed the effects of ridge cultivation with plastic mulching and the application rates of nitrogen. A paucity of research has been conducted on the multi-path migration, transformation, and loss of crop system nitrogen cycles under the interaction of these two factors, particularly in the context of forage crops. Alfalfa (*Medicago sativa* L.), the world’s most widely cultivated high-quality leguminous forage, is often referred to as the “King of Forage” [32,33,34]. Furthermore, alfalfa possesses extensive root systems, abundant foliage, and high ground cover, thereby enhancing soil fertility and reducing soil erosion, thus offering significant ecological value [35,36]. In principle, alfalfa has the capacity to satisfy its nitrogen requirements through nitrogen fixation by rhizobia. However, in production practice, the application of nitrogen fertilizer remains widespread globally in order to ensure the establishment of seedlings and the subsequent regrowth of vegetation following the harvesting of crops. The study hypothesizes that ridge cultivation with plastic mulching may interact with nitrogen application levels by regulating soil water and thermal conditions, jointly influencing nitrogen cycling and fate in alfalfa systems. The objective of the present study is to ascertain the findings after two years of field experiments. The following three objectives are to be pursued: (1) elucidation of the spatiotemporal patterns of soil NO_3_^−^–N and NH_4_^+^–N; (2) revelation of the characteristics of N_2_O emission and reduction potential in alfalfa grasslands; and (3) determination of optimal cropping patterns and nitrogen application thresholds for achieving efficient nitrogen balance within the system. The objective of this research is to establish the theoretical foundations and practical references for the optimization of nitrogen management and the promotion of green, sustainable development in alfalfa production.

## 2. Results

### 2.1. Nitrogen Uptake and Nitrogen Accumulation in Biomass

#### 2.1.1. Distribution of Inorganic Nitrogen Content in Soil Profiles

**Figure 1:**
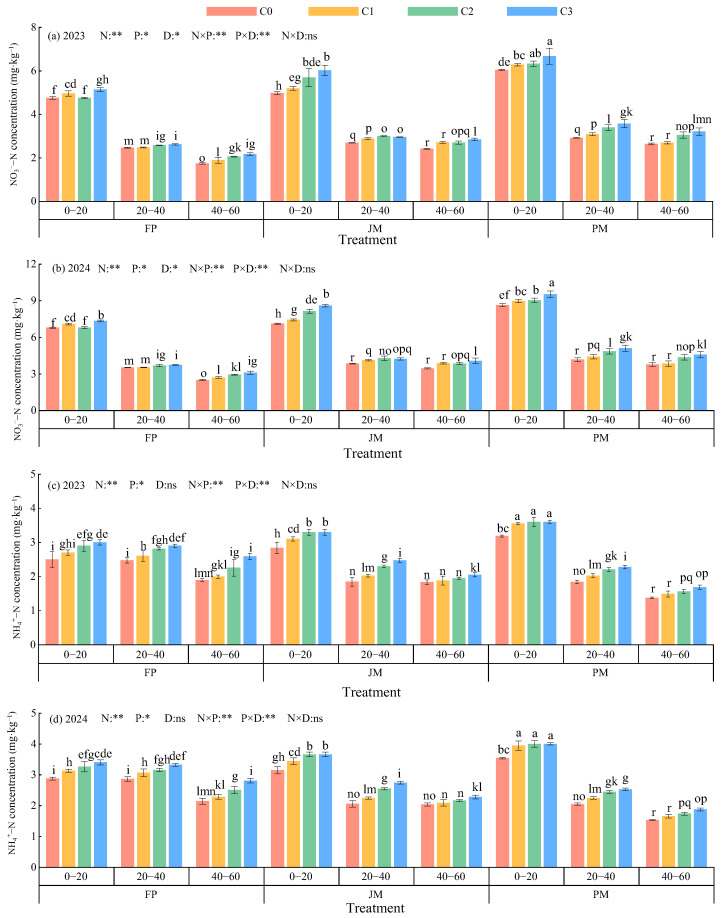
Effects of cropping patterns and nitrogen application rates on inorganic nitrogen content in soil profiles. (**a**,**b**) show the distribution of soil nitrate nitrogen content in 2023 and 2024, respectively. (**c**,**d**) show the distribution of soil ammonium nitrogen content in 2023 and 2024, respectively. N: Nitrogen rate; P: Plant pattern; D: Soil depth. ** indicates extremely significant difference (*p* < 0.01); * indicates significant difference (*p* < 0.05); ns indicates no significant difference (*p* > 0.05). Different lowercase letters indicate significant differences (*p* < 0.05).

The planting patterns and nitrogen application rates employed significantly influenced the soil NO_3_^−^–N and NH_4_^+^–N content in the 0–60 cm soil profile (Figure 1). The results demonstrated that both nitrogen forms exhibited a pattern of PM > JM > FP, with an increase at higher nitrogen application rates and a decrease at greater soil depth. In the context of diverse cropping systems, the C3 treatments exhibited higher concentrations of NO_3_^−^–N and NH_4_^+^–N in comparison to the C0, C1, and C2 treatments. Notably, the PMC3 treatments demonstrated the most substantial average content across all soil layers and on a comprehensive scale.

#### 2.1.2. Soil Inorganic Nitrogen Reserves

**Figure 2:**
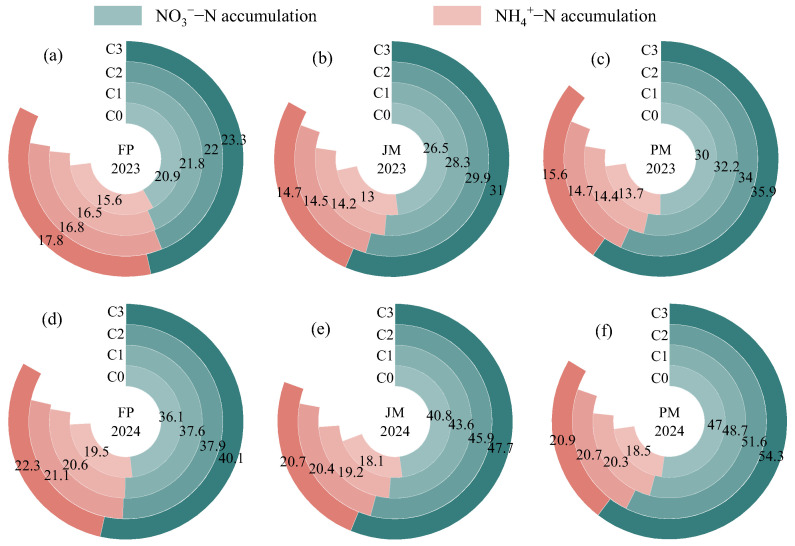
The effects of cropping systems and nitrogen application rates on soil inorganic nitrogen reservoirs in the 0–60 cm soil profile. (**a**–**c**) show the residual levels of soil inorganic nitrogen under FP, JM and PM treatments in 2023, respectively. (**d**–**f**) show the residual levels of soil inorganic nitrogen under FP, JM and PM treatments in 2024, respectively.

Soil NO_3_^−^–N and NH_4_^+^–N reserves in the 0–60 cm profile was significantly affected by the experimental year, nitrogen application rate, and their interaction (*p* < 0.01, Table 1). Compared to 2023, soil NO_3_^−^–N and NH_4_^+^–N reserves in 2024 was increased by 51.51–72.41% and 25.00–42.86%, respectively, demonstrating a clear upward trend with higher nitrogen application rates (Figure 2). During the 2024 growing season, soil NO_3_^−^–N accumulation generally followed the order of PM > JM > FP, with the highest values (35.9–38.5 mg·kg^−1^ and 54.3–58.3 mg·kg^−1^) being recorded in the PMC3 treatment. In contrast, no significant differences in soil NH_4_^+^–N reserves were observed among the different cropping patterns. The observed annual effect is primarily attributable to differences in precipitation during the growing seasons of 2024 and 2023, which altered downward leaching fluxes of soil nitrogen, resulting in the retention of inorganic nitrogen within the 0–60 cm soil profile. Elevated nitrogen application levels further amplified this ‘retention’ effect by increasing external inputs, thereby producing a significant “year × nitrogen application” interaction.

### 2.2. Nitrogen Uptake and Biomass Accumulation in Alfalfa Plants

#### 2.2.1. Plant Nitrogen Uptake

**Figure 3:**
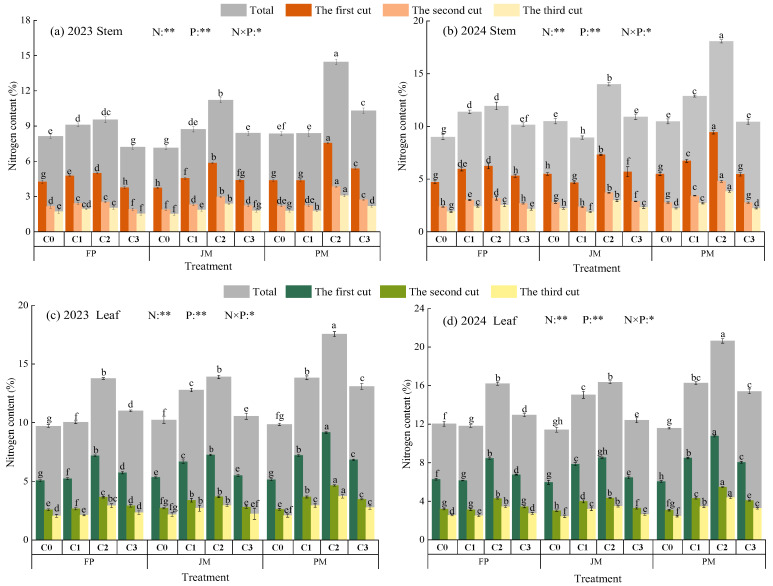
Effects of Planting Patterns and Nitrogen Application Rates on Nitrogen Uptake in Alfalfa Plants. (**a**,**b**) show the nitrogen content in plant stems for 2023 and 2024, respectively. (**c**,**d**) show the nitrogen content in plant leaves for 2023 and 2024, respectively. N: Nitrogen rate; P: Plant pattern. ** indicates extremely significant difference (*p* < 0.01); * indicates significant difference (*p* < 0.05). Different lowercase letters indicate significant differences (*p* < 0.05).

Nitrogen uptake in alfalfa plants was significantly influenced by nitrogen application rate, cropping system, and their interaction (*p* < 0.05, Figure 3). During the two-year growing season, leaf nitrogen content was recorded in the ranges of 2.05–9.17% and 2.42–10.78%, while stem nitrogen content varied between 1.52 and 7.55% and 1.89–9.44%. At equivalent nitrogen application rates, nitrogen uptake in both leaves and stems followed the consistent pattern of PM > JM > FP, with a decreasing trend observed as crop rotation progressed. Under identical cropping patterns, leaf and stem nitrogen uptake initially increased and subsequently decreased with rising nitrogen application rates, reaching maximum values under C2 conditions. Under the combined effects of cropping pattern and nitrogen application rate, the PMC2 treatment was identified as having the most favorable impact on promoting plant nitrogen uptake.

#### 2.2.2. Nitrogen Accumulated in Plant Biomass

**Figure 4:**
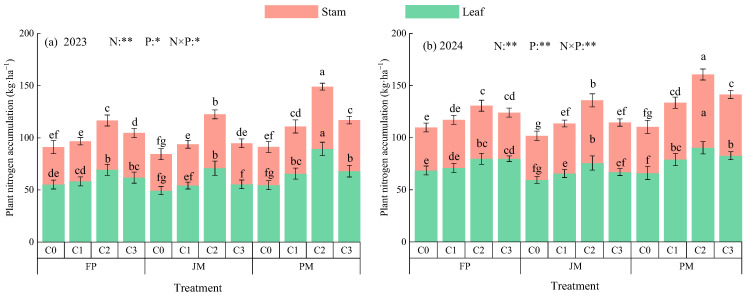
Effects of Planting Pattern and Nitrogen Application Rate on Nitrogen Accumulation in Alfalfa Plant Biomass. (**a**,**b**) show the nitrogen accumulation in the above-ground biomass of alfalfa plants in 2023 and 2024, respectively. N: Nitrogen rate; P: Plant pattern. ** indicates extremely significant difference (*p* < 0.01); * indicates significant difference (*p* < 0.05). Different lowercase letters indicate significant differences (*p* < 0.05).

Nitrogen accumulation in alfalfa plants was significantly affected by nitrogen application rates, planting patterns, and their interactions, with a progressive increase observed over the establishment period (Figure 4). Under consistent nitrogen application levels, the average plant biomass nitrogen accumulation in the PM treatment ranged from 100.74 to 154.31 kg·ha^−1^, exceeding values in FP (100.36–120.12 kg·ha^−1^) and JM (92.99–128.61 kg·ha^−1^) by 0.38–36.85% and 8.33–26.97%, respectively. For a given planting pattern, nitrogen accumulation was ordered as C2 > C3 > C1 > C0. Specifically, the C2 treatment resulted in average increases of 28.15–75.51% compared to C0, 20.37–44.68% over C1, and 11.56–36.87% relative to C3. Consequently, the PMC2 treatment was identified as the most effective in promoting nitrogen accumulation in alfalfa plants.

#### 2.2.3. Yield in Alfalfa Plants

**Figure 5:**
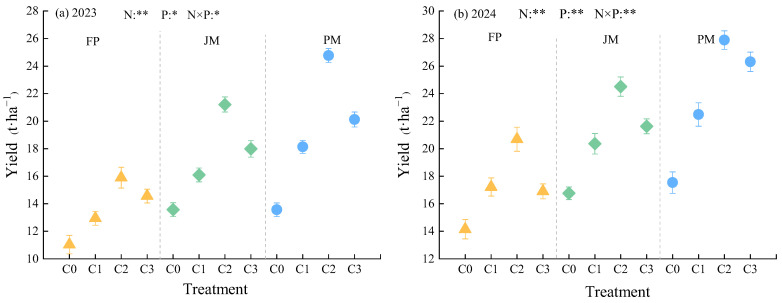
Effects of Planting Pattern and Nitrogen Application Rate on Yield in Alfalfa Plants. (**a**,**b**) show the alfalfa yield for 2023 and 2024, respectively. N: Nitrogen rate; P: Plant pattern. ** indicates extremely significant difference (*p* < 0.01); * indicates significant difference (*p* < 0.05). Error bars and scatter points represent the 95% confidence interval and yield values, respectively. Data represent the average of three replicates.

The planting patterns and nitrogen application rates employed had a significant impact on alfalfa yield. A comprehensive analysis reveals a 12.63% increase in alfalfa yield, reaching 32.99% in 2024 as compared to the 2023 yield (Figure 5). A comparison of alfalfa yield under ridge cultivation with plastic mulch with that of the FP treatment revealed an increase of 18.25% to 55.69% in 2023 and 22.92% to 55.77% in 2024. Alfalfa yield exhibited an initial increase, subsequently followed by a decrease in response to elevated nitrogen application rates, reaching a maximum under the C2 treatment. In comparison with C0, the application of elevated nitrogen rates resulted in an average increase in alfalfa yield of 6.58%, reaching 31.59%. During the two growing seasons, the PMC2 treatment resulted in the highest alfalfa production, at 24.76 t·ha^−1^ (2023) and 27.89 t·ha^−1^ (2024), respectively.

### 2.3. Grassland N_2_O Emissions

#### 2.3.1. Emission Flux

**Figure 6:**
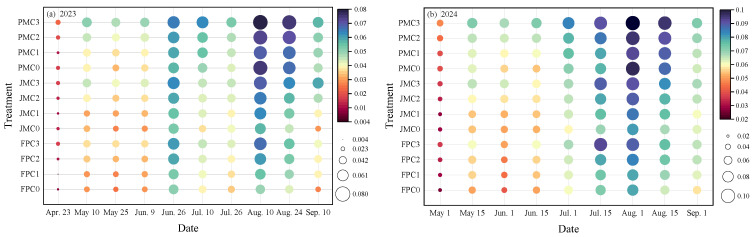
Effects of planting patterns and nitrogen application rates on N_2_O emission fluxes from grasslands. (**a**,**b**) show the N_2_O emission fluxes from grassland under different experimental treatments in 2023 and 2024, respectively.

Nitrous oxide (N_2_O) emission fluxes from alfalfa grasslands were measured under varying cropping systems and nitrogen application rates during the 2023 and 2024 growing seasons. In 2023, fluxes ranged from 0.001 to 0.078 mg·m^−2^·h^−1^, with peak emissions observed in June and August. During the 2024 growing season, fluxes varied between 0.001 and 0.010 mg·m^−2^·h^−1^, with peaks occurring in July and August (Figure 6). Within the same cropping system, the average N_2_O emission flux under the C3 treatment was found to be 3.51–91.27% higher than C0, 6.24–80.04% higher than C1, and 2.93–24.94% higher than C2. When the nitrogen application rate was held constant, the average N_2_O emission fluxes in the PM and JM systems were increased by 4.56–87.47% and 3.23–50.04%, respectively, relative to the FP system. Among all treatments, the highest N_2_O emission fluxes were recorded in the PMC3 treatment, with values ranging from 0.027 to 0.078 mg·m^−2^·h^−1^ in 2023 and 0.047 to 0.100 mg·m^−2^·h^−1^ in 2024. In contrast, the lowest fluxes were observed under the FPC0 treatment, ranging from 0.011 to 0.052 mg·m^−2^·h^−1^ in 2023 and 0.025 to 0.079 mg·m^−2^·h^−1^ in 2024.

#### 2.3.2. Total Emissions

**Figure 7:**
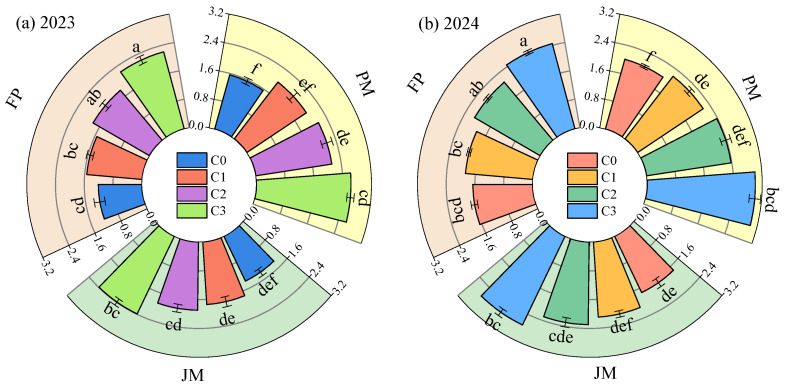
Effects of planting patterns and nitrogen application rates on total N_2_O emissions from grasslands. (**a**,**b**) show the total N_2_O emissions from grasslands under different experimental treatments in 2023 and 2024, respectively. Different lowercase letters indicate significant differences (*p* < 0.05).

Nitrogen application rates, cropping systems, and their interactions were found to significantly influence total N_2_O emissions from alfalfa grasslands (Figure 7). Total N_2_O emissions in 2024 were 18.54% to 22.38% higher than those recorded in 2023. When nitrogen application rates were held constant, total N_2_O emissions across cropping systems followed the order: PM (1.56–3.01 kg·ha^−1^) > JM (1.38–2.73 kg·ha^−1^) > FP (1.21–2.45 kg·ha^−1^). Under the same planting pattern, total N_2_O emissions increased with rising nitrogen application rates. Compared to the C0 treatment, the C1, C2, and C3 treatments exhibited average increases in total N_2_O emissions of 4.76–6.27%, 10.36–13.24%, and 18.21–22.30%, respectively. Results from Trial 2a demonstrated that the PMC3 treatment yielded the highest total N_2_O emissions, exceeding those of other treatments by an average of 22.86% to 28.92%.

### 2.4. Plant–Soil–Atmosphere System Nitrogen Balance

The nitrogen balance results for the alfalfa plant–soil–atmosphere system under different cropping patterns and nitrogen application rates (Table 2) indicate that among the three cropping patterns, C0 and C1 exhibited soil nitrogen deficiency, C3 showed severe soil nitrogen surplus, while C2 maintained relatively optimal soil nitrogen conditions. with apparent nitrogen balances ranging from 1.84 to 53.42 kg·ha^−1^. At the C2 level, both the apparent nitrogen balance and apparent nitrogen loss rate of PM were lower than those of FP and JM. Therefore, the soil nitrogen balance status under the PMC2 treatment was optimal, making it a suitable nitrogen application pattern for alfalfa cultivation.

## 3. Discussion

### 3.1. Effects of Nitrogen Application Patterns on Inorganic Nitrogen Content and Reserves in Soil Profiles

Nitrate nitrogen (NO_3_^−^–N) and ammonium nitrogen (NH_4_^+^–N) represent readily absorbable nitrogen forms that are directly utilized by crops, playing an essential role in the synthesis of amino acids, proteins, and other vital plant compounds [37,38]. The implementation of appropriate nitrogen application patterns during cultivation has been shown to significantly alter soil nitrogen transformation through the improvement of soil hydrothermal conditions, enhancement of soil respiration, and stimulation of microbial activity [10,22]. In the present study, soil NO_3_^−^–N and NH_4_^+^–N concentrations within the 0–60 cm soil profile were observed to decrease progressively with increasing soil depth (Figure 1), which is consistent with findings reported by Zupanic et al. and Lei et al. [39,40]. This vertical distribution pattern is primarily attributed to the surface soil layer, characterized by adequate oxygen availability, favorable temperatures, and abundant organic matter, which promotes the efficient conversion of NH_4_^+^ to NO_3_^−^ through the activity of ammonium-oxidizing and nitrifying bacteria [41]. With increasing soil depth, oxygen concentration gradually declines, temperatures decrease, and organic nitrogen sources become limited, collectively suppressing the activity of aerobic nitrifying bacteria and associated enzymatic reactions [42]. Furthermore, the anionic nature of NO_3_^−^–N prevents its adsorption by soil colloids, allowing continuous leaching to deeper soil layers through gravitational water movement, thereby reducing nitrogen retention in subsurface horizons [43]. The NO_3_^−^–N and NH_4_^+^–N contents of the soil exhibited higher levels in the presence of PM. (Figure 1), a pattern predominantly influenced by the differential regulation of soil microenvironments under these cropping systems. Compared to conventional FP, the improved soil hydrothermal regime under ridge-film mulching promotes the decomposition of soil organic matter, enhances soil enzyme activity and nutrient availability, and consequently accelerates soil nitrogen transformation and cycling. Alfalfa can be harvested multiple times per year, utilizing the spring, summer, and autumn growing seasons. In comparison with biodegradable mulch film, conventional mulch film has been shown to create a consistently stable “warm-humid and aerobic” microenvironment throughout the entire growth cycle of alfalfa. However, the utilization of biodegradable mulch film is accompanied by the development of microcracks as it undergoes degradation over time, resulting in diminished soil thermal and moisture stability. As the crop growth cycle progresses, the conditions approach those found in unmulched soil [11]. In a separate study on maize, Huang et al. [44] reported that ridge cultivation with biodegradable mulch slightly increased the soil carbon-to-nitrogen ratio compared to conventional plastic mulch, thereby facilitating more stable nitrogen retention. The discrepancy between these findings may be explained by crop-specific physiological characteristics. Alfalfa, which can be harvested three or more times annually with a growth period spanning spring to autumn [45], maintains a consistently stable “warm-humid-aerobic” microenvironment under PM throughout its entire growth cycle [46]. In contrast, biodegradable mulches gradually deteriorate over time, developing micro-cracks that approximate an uncovered condition during later growth stages. Maize, as a gramineous crop, exhibits particular sensitivity to water and temperature fluctuations, where elevated temperatures may induce premature root senescence. The progressive degradation of biodegradable mulch in later growth stages helps mitigate high temperature stress, thereby benefiting soil carbon and nitrogen equilibrium [47]. Ridge cultivation with mulch was found to significantly affect NO_3_^−^–N accumulation while demonstrating no substantial impact on NH_4_^+^–N accumulation relative to conventional flat planting (Figure 2). This differential response may be explained by the reduced vertical water permeability under ridge-mulch systems, which consequently diminishes NO_3_^−^–N leaching [41]. Additionally, the physical barrier created by the mulch film impedes ammonia volatilization at the soil–atmosphere interface, leading to more pronounced alterations in NO_3_^−^–N accumulation. In contrast, NH_4_^+^–N is readily adsorbed and stabilized by soil colloids, exhibiting significantly lower mobility [10].

The present study established a correlation between the content of NO_3_^−^–N and NH_4_^+^–N and the nitrogen application rate within the same cropping system. (Figure 1), corroborating observations by Spyrou et al. [48] in purslane and Jing et al. [49] in maize-soybean intercropping systems. This pattern likely results from exogenous nitrogen inputs augmenting soil inorganic nitrogen pools, thereby supplying additional substrates for nitrification processes [23]. High nitrogen application stimulates microbial activity, accelerating mineralization and nitrification rates, and consequently elevating soil NO_3_^−^–N and NH_4_^+^–N concentrations [22,23]. However, under actual field conditions, not all applied nitrogen is utilized by crops; significant portions are lost through leaching, volatilization, and other pathways [3]. The present study established that the application of elevated levels of nitrogen to soil significantly increases nitrogen accumulation in the soil (Figure 2). This phenomenon can be attributed to the application of nitrogen that exceeds the alfalfa’s actual demand, in conjunction with the concurrent conversion, fixation, and leaching processes during the growing season. Consequently, excess nitrogen accumulates in the soil profile as inorganic compounds. Concurrently, microbial activity converts this nitrogen into nitrate and ammonium forms. Nitrate nitrogen, being less readily adsorbed by soil, accumulates primarily in the 0–60 cm soil layer under conditions lacking intense leaching. This ultimately manifests as a significant increase in soil inorganic nitrogen accumulation with rising nitrogen application rates [22].

### 3.2. Effects of Nitrogen Application Patterns on Nitrogen Uptake and Nitrogen Accumulation in Alfalfa Biomass

The ridge-planting mulching system effectively regulates soil moisture, nutrient availability, aeration, and thermal conditions, thereby improving soil nitrogen availability. In this study, at equivalent nitrogen application rates, alfalfa leaves and stems grown under ridge-mulching exhibited higher nitrogen uptake than those grown under conventional flat-bed cultivation. Compared to FP, nitrogen accumulation in alfalfa biomass under PM and (JM increased by an average of 2.75% to 51.39% and 7.30% to 43.82%, respectively. (Figure 3 and Figure 4). This improvement can be primarily attributed to two key effects of conventional plastic film mulching on ridges. First, it significantly enhances the mineralization of soil organic nitrogen, thereby increasing soil nitrogen supply [10,12]. Second, it physically reduces nitrogen loss by inhibiting ammonia volatilization, thereby retaining more nitrogen in the root zone and improving plant nitrogen uptake efficiency [13]. In contrast, biodegradable mulch, while performing comparably to conventional mulch during early growth stages, gradually degrades in mid-to-late growth phases. This degradation leads to reduced water retention, nutrient preservation, and thermal regulation effects, resulting in an overall efficacy slightly lower than that of conventional mulch [50]. Under traditional flat cultivation, soil compaction is more likely to occur, hydrothermal conditions are less stable, and multiple pathways for nitrogen loss exist [51]. Notably, Zhang et al. [52] reported in their study on red clover that ridge-covered biodegradable mulch outperformed conventional plastic mulch. This discrepancy may be explained by the different research focuses: Zhang et al. emphasized ecological benefits, whereas the present study evaluates planting patterns from the perspective of synergistically enhancing both alfalfa yield and ecological benefits under a nitrogen balance framework. The substantial yield gains from conventional plastic mulch have the potential to fully offset and even exceed the costs associated with its residual film recovery. Moreover, when considering the long-term adaptability of crop production, ridge cultivation with conventional plastic mulch has been shown to offer greater reliability in maintaining optimal soil and water conditions during critical crop growth stages. Conversely, ridge-covered biodegradable films undergo degradation during the mid-to-late stages of decomposition, resulting in a substantial reduction in their water retention and weed suppression properties [52]. In the current study, nitrogen content in alfalfa leaves and stems was observed to decrease with successive harvest rotations (Figure 3), a trend consistent with findings reported by Li et al. [53]. This pattern may be attributed to the growth characteristics of different alfalfa rotations. The first harvest grows under cooler spring conditions with a longer growth cycle, while the second and third harvests develop during warmer summer months with accelerated growth rates. Under these conditions, the accumulation rates of carbohydrates (e.g., cellulose and lignin) may exceed those of nitrogen, leading to a “dilution effect” that reduces nitrogen concentration in plant tissues [54]. Furthermore, repeated harvesting depletes nutrient reserves in the root system, gradually weakening its nutrient uptake capacity [54].

Nitrogen serves as a fundamental element for the synthesis of proteins, amino acids, and other essential compounds in alfalfa, with its content closely linked to forage nutritional value [55]. In this study, nitrogen content was consistently higher in leaves than in stems (Figure 3). This difference reflects their distinct physiological roles: leaves are the primary sites for high-nitrogen-demand processes such as photosynthesis, protein synthesis, and nitrogen assimilation, and are rich in nitrogenous compounds including proteins, nucleic acids, and chlorophyll [56]. Stems, on the other hand, function mainly in structural support and transport, composed largely of cellulose and lignin, which contain relatively low nitrogen levels [57]. A well-designed nitrogen application regime under plastic film mulching can effectively optimize soil nitrogen supply and enhance plant nitrogen uptake efficiency. Under identical cropping systems, both nitrogen uptake and accumulation in alfalfa biomass exhibited a trend of initial increase followed by a decrease with rising nitrogen application rates, reaching maximum values at the C2 level (Figure 4). These results align with previous studies by Qiao et al. [58] and Yu et al. [59]. This phenomenon can be attributed to the fact that, at low nitrogen application rates, additional nitrogen fertilizer effectively alleviates nitrogen limitation in plants. This, in turn, promotes chlorophyll synthesis and photosynthesis, thereby increasing nitrogen concentration and accumulation within the plant. However, it has been demonstrated that when the application of nitrogen exceeds the crop’s absorption threshold, there is a decline in nitrogen fertilizer utilization efficiency. Excess nitrogen enters groundwater through leaching, is converted into nitrogen gas or the potent greenhouse gas nitrous oxide via denitrification, and enters the environment through loss pathways such as soil fixation and biological fixation, leading to nutrient loss and environmental pollution [30,35]. Furthermore, the presence of antagonistic ions in soil has been demonstrated to impede the absorption of potassium, calcium, magnesium, and other elements by crops, resulting in a nutritional imbalance [28]. In the context of alfalfa, the presence of excessive inorganic nitrogen in the soil has been shown to result in the suppression of nitrogenase activity in rhizobia. This, in turn, leads to a disruption of alfalfa’s physiological metabolism and a consequent reduction in nitrogen accumulation [28].

### 3.3. Effects of Nitrogen Application Patterns on N_2_O Emissions from Alfalfa Grasslands

Nitrous oxide (N_2_O) is the third most significant greenhouse gas and the predominant ozone-depleting substance globally [5]. In this study, under equivalent nitrogen application rates, alfalfa grasslands demonstrated the highest N_2_O emission flux and cumulative emissions, followed by JM, while FP showed the lowest values (Figure 6 and Figure 7). This finding is consistent with observations reported by Miao et al. [60] in dryland potato systems. Ridges mulched with conventional plastic create a microenvironment with elevated temperature and stable moisture, functioning as a sustained “incubator” for nitrification and denitrification processes. In contrast, ridges with biodegradable mulch experience gradual film degradation during the mid-to-late growing season, which diminishes their moderating effect on soil temperature and moisture. Conventional flat planting, characterized by larger diurnal temperature fluctuations and alternating drying and wetting cycles, tends to suppress the rates of soil nitrification and denitrification [10,50]. Furthermore, the surface soil in ridged mulched plots contained significantly higher concentrations of NO_3_^−^–N and NH_4_^+^–N than flat-bed plots, providing a substantial substrate reservoir that supports elevated N_2_O production. The soil profile under ridge mulching may also develop a three-dimensional gradient, with an aerobic surface layer and a microaerophilic ridge base, creating additional hotspots for N_2_O generation. In contrast, the more uniform oxygen distribution in traditional flat-bed plots limits denitrification potential [61]. Cumulative soil N_2_O emissions in this study increased with nitrogen application rate. Compared to the C0 treatment, total N_2_O emissions under C1, C2, and C3 increased by 4.76–6.27%, 10.36–13.24%, and 18.21–22.30%, respectively (Figure 7), which aligns with the findings of Liu et al. [62]. Nitrogen application regulates both the microbial processes and substrate availability involved in N_2_O production. When nitrogen input exceeds crop demand, the surplus remains in the soil and continuously stimulates microbial activity, thereby promoting N_2_O emission [28]. Therefore, optimizing nitrogen management to maintain soil inorganic nitrogen below the threshold for microbial conversion is crucial for mitigating farmland N_2_O emissions.

Peak N_2_O emission periods can contribute to approximately 50% of annual farmland emissions [5]. In this study, N_2_O emission fluxes from alfalfa ranged from 0.001 to 0.078 mg·m^−2^·h^−1^ in 2023 and from 0.001 to 0.010 mg·m^−2^·h^−1^ in 2024, with emission peaks occurring mainly between early July and mid-August (Figure 6). This pattern is consistent with studies on peanut fields by Zhao et al. [63] and sunflower fields by Chen et al. [64]. The concentration of emission peaks during this period is likely attributable to high summer temperatures in July and August, which significantly enhance microbial activity. This warm period often coincides with seasonal rainfall or intensive irrigation, increasing soil moisture and facilitating the formation of anaerobic microsites. These combined factors stimulate nitrification and denitrification, leading to seasonal N_2_O emission maxima [65]. Therefore, it is crucial to consider that, although PM management improves nitrogen use efficiency and crop productivity, it may also increase the risk of N_2_O emissions. Consequently, the environmental trade-offs must be carefully considered during implementation.

### 3.4. Limitations and Future Directions

In order to calculate the soil–plant–atmosphere nitrogen balance for alfalfa, the present study considered nitrogen input (N_input_) to comprise applied nitrogen and pre-sowing soil inorganic nitrogen reserves in the 0–60 cm layer. However, biological nitrogen fixation—the largest ‘hidden nitrogen input’ in alfalfa systems—was not incorporated into nitrogen input calculations. Furthermore, the losses of nitrogen through leaching and NH_3_ volatilization were not fully accounted for, which may have resulted in “pseudo-deficiencies” in the low-nitrogen treatment groups. It is therefore recommended that future studies incorporate additional measurement methods with a view to accurately defining nitrogen application thresholds for alfalfa grasslands. This would help to avoid underestimation of potential risks relating to leaching and gaseous loss.

## 4. Materials and Methods

### 4.1. Experimental Site Overview

**Figure 8:**
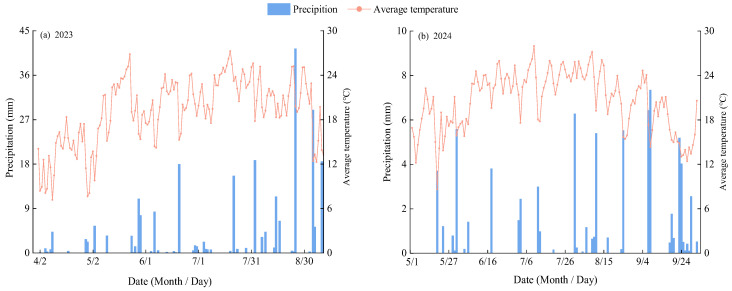
Daily precipitation and temperature distribution during the alfalfa growing season. (**a**,**b**) show the daily precipitation and temperature variations in the experimental areas for 2023 and 2024, respectively.

The field experiment was conducted at the Jingtaichuan Electric Power Lift Irrigation Water Resources Utilization Center Irrigation Experiment Station (37°23′ N, 104°08′ E; 2028 m altitude) in Gansu Province, during the alfalfa growing seasons (April–October) of 2023 and 2024. The region has a multi-year average precipitation of 191.6 mm, evaporation of 2761 mm, sunshine duration of 2652 h, solar radiation of 6.18 × 10^5^ J·cm^−2^, mean temperature of 8.5 °C, and a frost-free period of 191 days. The soil at the experimental site is a sandy loam with a dry bulk density of 1.45 g·cm^−3^, a maximum field capacity of 24.1% (mass water content), and a pH of 8.11. In the 0–30 cm soil layer, the organic matter, total nitrogen, total phosphorus, and total potassium contents were 6.09, 1.62, 1.32, and 34.03 g·kg^−1^, respectively, while the available nitrogen, available phosphorus, and available potassium contents were 74.51, 26.31, and 173 mg·kg^−1^, respectively. During the experimental growing seasons, the average daily temperature and total precipitation were 20.58 °C and 186.15 mm in 2023, and 12.28 °C and 361.35 mm in 2024 (Figure 8).

### 4.2. Experimental Design

**Figure 9:**
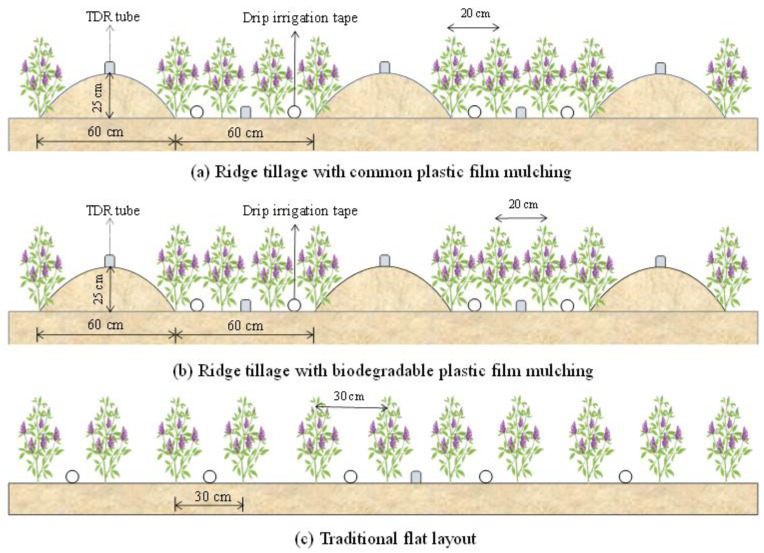
Schematic diagram of alfalfa cultivation patterns.

The field experiment utilized the ‘Longdong Purple Alfalfa’ cultivar. The experiment employed a completely randomized block design. A two-factor completely randomized design was employed, encompassing planting patterns (Figure 9) and nitrogen application rates (Table 3). The planting patterns consisted of conventional flat planting (FP), ridge planting with biodegradable film mulch (JM), and ridge planting with conventional plastic mulch (PM). Nitrogen was applied at four rates: 0, 80, 160, and 240 kg N ha^−1^, resulting in a total of 12 treatments, each replicated three times, the unit area is 42.9 m^2^ (5.5 m × 7.8 m). Alfalfa was sown on 7 April 2021, at a seeding rate of 22.5 kg ha^−1^. In plots treated with PM and JM, alfalfa was planted along the ridges and in the furrows after the trenches had been formed and the ridges created. Four rows were planted in each furrow, spaced 20 cm apart. In plots treated with FP, alfalfa was planted with 30 cm row spacing. Prior to the first regrowth each year, nitrogen fertilizer (controlled-release urea, 30% N, Shandong Jinzhengda Ecological Engineering Group Co., Ltd., Linyi, China), phosphorus fertilizer (calcium superphosphate, 16% P_2_O_5_) at 50 kg P_2_O_5_ ha^−1^, and potassium fertilizer (potassium chloride, 60% K_2_O) at 50 kg K_2_O ha^−1^ were applied once as a basal fertilizer. A drip irrigation system was installed with drip tapes spaced 60 cm apart, using emitters with a flow rate of 2 L h^−1^. Irrigation volumes were regulated and monitored using valves and water meters (accuracy: 0.001 m^3^) installed on the supply pipes. All irrigation and field management practices followed local standards for conventional grassland management.

### 4.3. Test Item and Method

#### 4.3.1. Soil NO_3_^−^–N and NH_4_^+^–N Content and Accumulation

After the third alfalfa harvest each year, soil samples were collected from the 0–60 cm soil layer (at 20 cm intervals) in each plot using an S-shaped sampling method with a soil auger. For ridged mulched plots, samples were taken from the exact center of the furrow. Soil samples were air-dried, thoroughly mixed, and sieved (2 mm). (5 g dry soil, soil-to-liquid mass ratio 1:10). Soil NO_3_^−^–N content (mg·kg^−1^) was determined using a UV-visible spectrophotometer (Beijing Puxi General Instrument Co., Ltd., Beijing, China, T6 New Century), while soil NH_4_^+^–N content (mg·kg^−1^) was measured via the indophenol blue colorimetric method.

The calculation formula for soil NO_3_^–^N (NH_4_^+^–N) accumulation (Nitrogen accumulation, *NR*, kg·ha^−1^) is Equation (1) [66]:

(1) $$\mathrm{NR}\;=\;\frac{\gamma_{i}h_{i}N_{i}}{10}$$

where *γ_i_* denotes the bulk density of the *i*th soil layer (g·cm^−3^), *h_i_* represents the depth of the *i*th soil layer (cm), and *N_i_* indicates the NO_3_^−^–N (NH_4_^+^–N) content of the *i*th soil layer (mg·kg^−1^).

#### 4.3.2. Alfalfa Yield and Nitrogen Content and Accumulation in Plants

(1) Yield

The yield of alfalfa was determined by means of the plot method. At the initial flowering stage of each crop, a 1 m^2^ (1 m × 1 m, this method is designed to perform three repeated samples) plot with uniform growth was selected from each subplot. The forage was harvested at a height of 5 centimeters above ground level, any weeds were removed, and the fresh forage weight was measured. The forage was then subjected to blanching at 105 °C for 0.5 h, after which it was dried at 75 °C until it reached a constant weight. Following a cooling period, the dry weight was measured in order to calculate the hay yield per unit area. This yield was then converted to a per unit area measurement (kg·ha^−1^).

(2) Plant Nitrogen Content and Accumulation

At the initial flowering stage of each alfalfa crop, 10 uniformly growing alfalfa plants were randomly selected from each plot for cutting. The stems and leaves were separated, blanched at 105 °C for 30 min, then dried at a constant temperature of 75 °C. After cooling, their dry weight was measured. The dried leaves and stems were separately pulverized using a small grinder and sieved through a 1 mm mesh. Following digestion with H_2_SO_4_−H_2_O_2_, the total nitrogen content of both leaves and stems was determined using a FOSS 2300 fully automated Kjeldahl nitrogen analyzer.

Alfalfa leaf (stem) nitrogen accumulation (kg·ha^−1^) = Leaf (stem) nitrogen content (%) × Dry matter yield per unit area of leaves (stems) (kg·ha^−1^). The sum of nitrogen accumulation in leaves and stems represents the total nitrogen accumulation in the alfalfa plant [67].

#### 4.3.3. Soil N_2_O Emissions

**Figure 10:**
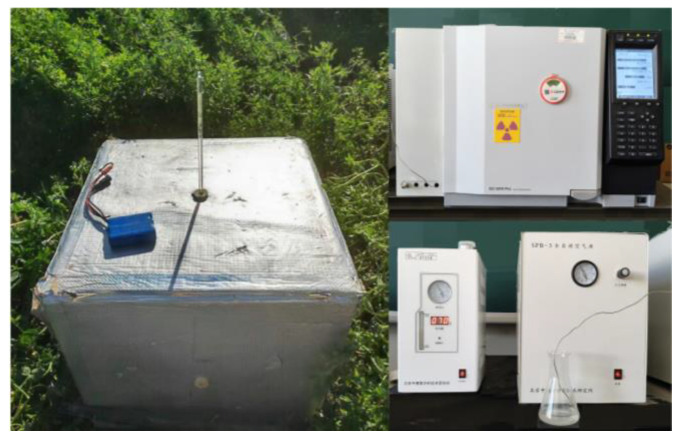
Sealed static dark box and meteorological chromatograph.

The soil N_2_O emission flux throughout the alfalfa growing season was quantified using the closed static chamber technique coupled with a gas chromatograph (Shimadzu GC-2010Pro, Shanghai Silicon Instruments Biochemical Technology Co., Ltd., Shanghai, China) (Figure 10). The chamber assembly comprised a removable PVC top (60 cm × 60 cm × 60 cm) covered with insulating material and a base (65 cm × 65 cm × 25 cm) designed with a water-sealing groove. The chamber top incorporated a sampling port linked to a three-way valve, an internal thermometer, and a small fan for air circulation. The base was permanently installed by inserting it 20 cm into the soil. Gas samples were collected from the center of flat-bed (FP) plots and from both ridges and furrows in ridge–furrow (JM, PM) systems (The N_2_O flux is calculated as a weighted average, based on the ratio of the areas at the ridge top and the trench bottom.). Post-fertilization sampling occurred at 15-day intervals, with control (unfertilized) plots sampled concurrently to maintain a consistent temporal dataset.

The calculation formula for N_2_O emission flux (F, kg·m^−2^·h^−1^) is Equation (2) [68]:

(2) $$F\;=\;\rho \;\times \;H\;\times \;\frac{d_{c}}{d_{t}}\;\times \;\frac{273}{(273+T)}\;\times \;60$$

where *ρ* is the density of N_2_O gas under standard conditions (*ρ* = 2 × 14/22.4 = 1.25), kg·m^−3^; *H* is the height of the chamber, m; $\frac{d_{c}}{d_{t}}$ is the rate of change of N_2_O concentration in the chamber over time during sampling, μL·L^−1^·min^−1^; *T* is the average temperature inside the chamber during sampling, °C.

The calculation formula for total N_2_O emissions (*f*, kg·ha^−1^) is Equation (3) [69]:

(3) $$f\;=\;\sum \frac{F_{i+1}\;+\;F_{i}}{2}\;\times \;t\;\times \;24\;\times \;10^{-5}$$

where *F* denotes the N_2_O emission flux, ug·m^−2^·h^−1^; *i* denotes the sampling frequency; *t* denotes the interval in days between the *i*th and *i* + 1th sampling events, d.

#### 4.3.4. Nitrogen Balance

Based on integrated soil–plant–atmosphere analysis, the apparent nitrogen balance (N_balance_) and apparent nitrogen loss rate (ANLR, %) of the alfalfa system. Nitrogen input (N_input_) comprised applied nitrogen and pre-sowing soil inorganic nitrogen reserves in the 0–60 cm layer. Nitrogen output (N_output_) included nitrogen accumulation in alfalfa above ground biomass, soil inorganic nitrogen accumulation in the 0–60 cm layer, and total N_2_O emissions.

The calculation formula for nitrogen balance (*N_balance_*, kg·ha^−1^) is Equation (4) [70]:

(4) $$N_{\mathrm{balance}}\;={\;N}_{\mathrm{input}}{\;-\;N}_{\mathrm{output}}$$

In the equation, *N_input_* represents nitrogen input (kg·ha^−1^), and *N_output_* represents nitrogen output (kg·ha^−1^).

Apparent Nitrogen Loss Rate (*ANLR*, %) represents the percentage of nitrogen applied to the soil that is not absorbed and utilized by alfalfa, relative to the total nitrogen applied to the soil. The calculation formula is Equation (5):

(5) $$\mathrm{ANLR}\;(\%)\;\;=\;\frac{N_{\mathrm{balance}}}{N_{i}}\;\times \;100$$

In the equation, *N_balance_* represents the apparent nitrogen balance (kg·ha^−1^), and *N_i_* denotes the nitrogen application rate (kg·ha^−1^) for treatment *i*.

### 4.4. Data Statistics and Analysis

Data were organized using Microsoft Excel 2016, and one-way and two-way ANOVA analyses were performed using SPSS 2.1.0. Graphs were generated using Origin 2021. Post hoc comparisons between treatments were conducted using the Least Significant Difference (LSD) test, with a significance level set at *p* = 0.05.

## 5. Conclusions

The analysis yielded the following key results: (1) Soil nitrate and ammonium nitrogen contents followed the order PM > JM > FP and increased with nitrogen application rate. (2) Nitrogen content and accumulation in alfalfa leaves and stems also adhered to the PM > JM > FP order, exhibiting a trend of initial increase followed by a decrease with rising nitrogen application, peaking at the C2 level (160 kg·ha^−1^). (3) Both N_2_O emission flux and cumulative emissions from the alfalfa pasture were highest under PM, followed by JM and FP, and showed an increasing trend with nitrogen application rate. (4) Nitrogen balance analysis revealed nitrogen deficits in C0 and C1 treatments, a severe surplus in C3, and a state near equilibrium in C2. The PMC2 treatment combination resulted in the lowest apparent nitrogen loss, recorded at 6.08% in 2023 and 1.15% in 2024. In summary, the combination of ridge-covered conventional plastic mulch with moderate nitrogen application resulted in reduced apparent nitrogen loss, significant nitrogen accumulation, and high yields. This approach offers a promising and environmentally friendly strategy for managing nitrogen in alfalfa cultivation.

## Figures and Tables

**Table 1:** Analysis of variance of soil inorganic nitrogen reserves under different cropping systems and nitrogen application rates (F-value).

Category	NO_3_^−^–N	NH_4_^+^–N
N	31.41 **	4.39 **
P	237.02 **	0.80 ns
Y	6100.46 **	96.03 **
N × P	1.66 ns	0.016 ns
N × Y	5.26 **	0.137 ns
P × Y	46.19 **	2.22 ns
N × P × Y	0.266 ns	0.043 ns

N: Nitrogen rate; P: Plant pattern; Y: Year. ** indicates extremely significant difference (*p* < 0.01); ns indicates no significant difference (*p* > 0.05). Prior to conducting the analysis of variance, Shapiro–Wilk tests were performed on the residuals of all data to assess normality, and Levene’s tests were conducted to evaluate homogeneity of variance.

**Table 2:** Effects of Nitrogen Application Patterns on Nitrogen Balance During the Growth Period of Alfalfa in 2023 and 2024.

Treatment		*N_input_* (kg·ha^−1^)			*N_output_* (kg·ha^−1^)						*N_balance_* (kg·ha^−1^)		*ANLR* (%)	
		N Rate	Initial Nitrogen Content		Soil Nitrogen Residue		Nitrogen Accumulation in Plants		N_2_O Emissions		2023	2024	2023	2024
			2023	2024	2023	2024	2023	2024	2023	2024				
FP	C0	0	40.03	56	36.5	55.63	91.01	109.72	1.21	1.66	−88.69	−111.01	—	—
	C1	80	44.96	59.6	38.3	58.26	96.65	117.05	1.54	1.88	−11.53	−37.59	−14.41	−46.99
	C2	160	47.6	61.8	38.8	59.01	113.57	120.66	1.81	2.11	53.42	40.02	33.39	25.01
	C3	240	50.13	69.1	41.1	62.4	104.57	126.01	2.21	2.45	142.25	118.24	59.279	49.27
JM	C0	0	43.17	58.3	39.5	58.97	84.25	101.74	1.38	1.73	−81.96	−104.14	—	—
	C1	80	45.97	63.1	42.5	62.87	93.76	113.51	1.74	2.09	−12.03	−35.37	−15.04	−44.21
	C2	160	48.3	68.2	44.4	66.28	121.48	135.74	1.89	2.29	40.53	23.89	25.33	14.93
	C3	240	52.87	71.5	45.7	68.4	94.69	114.48	2.39	2.73	150.09	125.89	62.54	52.45
PM	C0	0	42.8	62.7	43.7	65.53	91.16	110.32	1.56	2.01	−93.62	−115.16	—	—
	C1	80	45.67	69.2	46.6	69	110.88	133.52	1.99	2.19	−33.8	−55.51	−42.25	−69.39
	C2	160	48.6	77.1	48.7	72.3	148.03	160.58	2.14	2.38	9.73	1.84	6.08	1.15
	C3	240	56.03	79.5	51.5	75.27	116.96	141.39	2.63	3.01	124.94	99.83	52.06	41.59

**Table 3:** Experimental treatments.

Growing Pattern	Nitrogen Application Rate/(kg·ha^−1^)
FP	0 (C0)
	80 (C1)
	160 (C2)
	240 (C3)
JM	0 (C0)
	80 (C1)
	160 (C2)
	240 (C3)
PM	0 (C0)
	80 (C1)
	160 (C2)
	240 (C3)

## Data Availability

All data supporting this study are included in the article.
